# Salidroside attenuates neuronal ferroptosis by activating the Nrf2/HO1 signaling pathway in Aβ_1-42_-induced Alzheimer’s disease mice and glutamate-injured HT22 cells

**DOI:** 10.1186/s13020-022-00634-3

**Published:** 2022-07-04

**Authors:** Sixia Yang, Zeping xie, Tingting Pei, Yi zeng, Qiaowu Xiong, Hui Wei, Yong Wang, Weidong Cheng

**Affiliations:** 1 School of Traditional Chinese Medicine, Southern Medical University, Guangzhou, 510515 China; 2 Department of Pharmacy, Zhu Jiang Hospital, Southern Medical University, Guangzhou, 510515 China

**Keywords:** Alzheimer's disease, Salidroside, Ferroptosis, HT22 cells, Nuclear factor E2-related factor 2

## Abstract

**Background:**

Alzheimer’s disease (AD) is a neurodegenerative disease. Ferroptosis plays a critical role in neurodegenerative diseases. Nuclear factor E2-related factor 2 (Nrf2) is considered an important factor in ferroptosis. Studies have demonstrated that salidroside has a potential therapeutic effect on AD. The intrinsic effect of salidroside on ferroptosis is unclear. The purpose of this study was to investigate the protective effects and pharmacological mechanisms of salidroside on alleviating neuronal ferroptosis in Aβ_1−42_-induced AD mice and glutamate-injured HT22 cells.

**Methods:**

HT22 cells were injured by glutamate (Glu), HT22 cells transfected with siRNA Nrf2, and Aβ_1−42_-induced WT and Nrf2^−/−^AD mice were treated with salidroside. The mitochondria ultrastructure, intracellular Fe^2+^, reactive oxygen species, mitochondrial membrane potential, and lipid peroxidation of HT22 cells were detected. Malondialdehyde, reduced glutathione, oxidized glutathione disulfide, and superoxide dismutase were measured. The novel object recognition test, Y-maze, and open field test were used to investigate the protective effects of salidroside on Aβ_1−42_-induced WT and Nrf2^−/−^AD mice. The protein expressions of PTGS2, GPX4, Nrf2, and HO1 in the hippocampus were investigated by Western blot.

**Results:**

Salidroside increased the cell viability and the level of MMP of Glu-injured HT22 cells, reduced the level of lipid peroxidation and ROS, and increased GPX4 and SLC7A11 protein expressions. These changes were not observed in siRNA Nrf2 transfected HT22 cells. Salidroside improved the ultrastructural changes in mitochondria of HT22 cells and Aβ_1−42_-induced AD mice, but not in Aβ_1−42_-induced Nrf2^−/−^AD mice. Salidroside increased protein expression levels of GPX4, HO1, and NQO1 and decreased protein expression of PTGS2 in Aβ_1−42_-induced AD mice but not in Aβ_1−42_-induced Nrf2^−/−^AD mice.

**Conclusions:**

Salidroside plays a neuroprotective role by inhibiting neuronal ferroptosis in Aβ_1−42_-induced AD mice and Glu-injured HT22 cells, and its mechanism is related to activation of the Nrf2/HO1 signaling pathway.

**Graphical Abstract:**

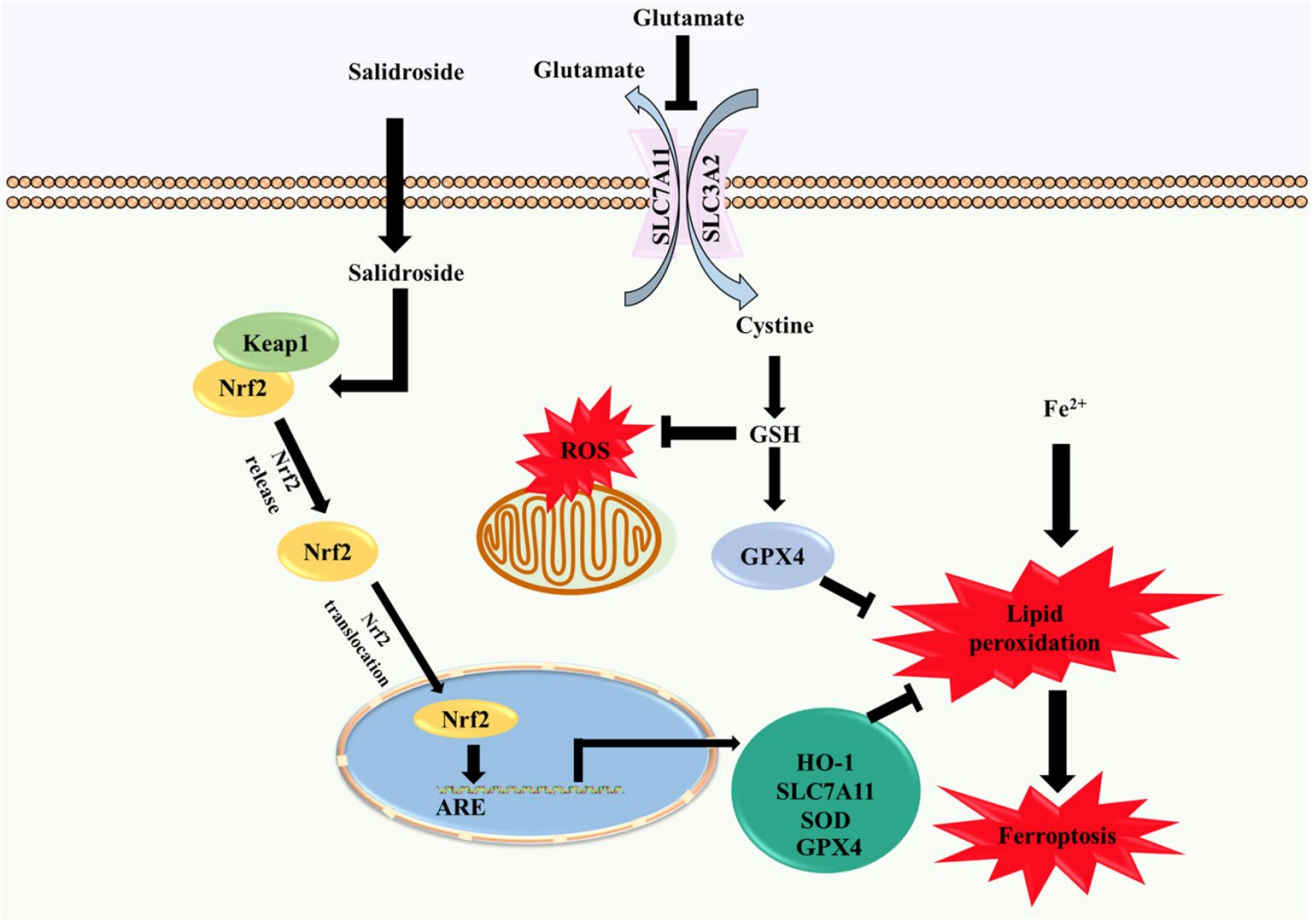

## Introduction

Alzheimer’s disease (AD) is a neurodegenerative disease. Symptoms include memory loss, mood changes, impaired judgment, difficulties in concentration, and a lack of planning, writing, and speech [1]. Previous studies have demonstrated that amyloid β (Aβ), Tau-containing neurofibrillary tangles, genetic susceptibility, and other risk factors play important roles in the development of AD [2]. The importance of increased oxidative stress was highlighted in the brains of patients with AD [3]. The levels of nuclear and mitochondrial DNA oxidation in the brains of AD patients were increased compared with those in age-matched controls, and mitochondrial DNA oxidation may contribute to the neurodegeneration of AD [4]. In AD pathogenesis, mitochondrial dysfunction is related to Aβ accumulation. Mitochondrial dysfunction and AD pathology are positive feedback, and the accumulation of damaged mitochondria in neurons is a hallmark of neurodegenerative disorders, including AD [5]. Massive neuronal cell death causes shrinkage throughout the cortex and hippocampus in advanced AD, but the etiology is not clear [6].

The accumulation of iron and lipid peroxidation was prevalent in neurodegenerative diseases, accompanied by the inhibition of glutathione (GSH) and glutathione peroxidase 4 (GPX4) [7]. Ferroptosis is a form of programmed cell death characterized by the iron-dependent accumulation of lipid peroxides. The unique hallmark of ferroptosis may be mitochondrial shrinkage [8]. Studies have suggested that ferroptosis plays a pivotal role in neurodegenerative disease. Ferroptosis inhibitors have been shown to exert cognitive and neuroprotective effects in animal models [9]. One of the regulatory pathways of ferroptosis is the GPX4-regulated ferroptosis pathway. It relays the cystine-glutamate antiporter (system xc-)-mediated uptake of cystine and thioredoxin reductase 1-dependent reduction of cystine to cysteine, which is used for GSH biosynthesis. GPX4 is an inhibitor of lipid peroxidation that degrades small molecular peroxides and relatively complex lipid peroxides and reduces them to their corresponding alcohols by mediating phospholipid hydroperoxides. Recycling of oxidized glutathione (GSSG) is achieved via glutathione-disulfide reductase using electrons provided by NADPH/H^+^ [10].

Studies have shown that glutamate neurotoxicity is involved in neurodegenerative diseases. Inhibition of the system xc- is involved in ferroptosis induced by high extracellular concentrations of glutamate [11]. Glutamate (Glu) treatment of HT22 cells leads to intracellular GSH depletion, leading to oxidative stress and ferroptosis [12]. In this study, HT22 cells injured by high concentrations of glutamate were used as an in vitro ferroptosis model.

Nuclear factor E2-related factor 2 (Nrf2) is a transcription factor and plays a key role in antioxidation. Under normal oxygen homeostasis, Nrf2 binds to Keap1 and is inactivated with the ubiquitination and degradation of the proteasome. Under oxidative stress, Nrf2 is released from the site bound to Keap1 and quickly transferred to the nucleus. It interacts with antioxidant response elements (ARE) to activate the transcription pathway, promote the transcription of downstream antioxidant genes and maintain cellular redox homeostasis [13]. The downstream genes of Nrf2 can regulate GSH production, involving solute carrier family 7 membrane 11 (SLC7A11), glutathione reductase (GSR), glutathione synthetase (GSS), glutamate-cysteine ligase, catalytic subunit (GCLC), regulate ROS detoxification, involved GPX4, heme oxygenase 1(HO1), NAD(P)H: quinone oxidoreductase 1(NQO1), and regulate iron metabolism, involved metallothionein-1G (MT1G), ferritin light chain (FTL), ferritin heavy chain (FTH1), solute carrier family 40 (iron-regulated transporter), member 1 (SLC40A1), ferrochelatase (FECH) [14, 15]. AD brains are prone to reactive oxygen species (ROS)-induced insults because of the decreased levels of Nrf2 and important antioxidant enzymes, including superoxide dismutase 1 (SOD1), catalase (CAT), and GPX4. Increasing the levels of antioxidant proteins through Nrf2 activation has been considered an alternative way to achieve neuroprotection [16].

*Rhodiola Rosea L.* has been used as an adaptogen in northern Europe and Russia and as a traditional herb in China. Research indicated that it can improve attention in cognitive function in fatigue [17]. Antioxidation, cholinergic regulation, anti-apoptosis, anti-inflammation, and improving brain metabolism are involved in *Rhodiola Rosea L.* improving learning and memory function [18]. There are many domestic and international studies on the chemical constituents of *Rhodiola Rosea L*. The main pharmacologically active ingredients isolated from various *Rhodiola Rosea L.* are salidroside and its aglycone (tyrosol), rosavin, pyridine, rhodopsin, and rhodionin [19]. Salidroside, a p-hydroxyphenyl-β-glucoside compound, is distributed in all parts of the plant and has various biological activities and a wide spectrum of pharmacological properties [20]. Studies have indicated that salidroside has neuroprotective effects in an AD model by protecting the damaged synapses of neurons [21], regulating the microbiota-gut-brain axis, modulating inflammation [22], reducing PC12 cell apoptosis [23], exerting antioxidant activity, and protecting mitochondrial function [24]. Salidroside may have potential as a treatment for AD [20]. Salidroside was not genotoxic at the dose of 1.5 g/kg/d administration to mice after continuous administration for 3 days [25]. Studies have shown that salidroside is a safe substance, and no obvious adverse reactions found in pre-clinical and clinical trials indicated that salidroside is promising as a common clinical drug [26].

Studies have shown that salidroside can reduce oxidative stress and apoptosis of KGN cells by activating Nrf2 [27], exerting anticonvulsant and neuroprotective effects in epileptic rats by activating Nrf2 are signaling pathway [28], and regulating Nrf2 after pMCAO to reduce neuroinflammation and nerve injury [29]. Therefore, the activation of Nrf2 may be involved in the neuroprotective effect of salidroside. Although many studies have reported the neuroprotective effects of salidroside on neurodegenerative diseases, however, the effects of salidroside and *Rhodiola Rosea L*. on cellular iron homeostasis, iron regulatory protein were unclear. There were few studies reported the effects of salidroside and *Rhodiola Rosea L*. on neurons ferroptosis in AD. To further explore the neuroprotective effects and pharmacological mechanisms of salidroside, this study proposed that salidroside played a neuroprotective role in Aβ_1–42_-induced AD mice and Glu-injured HT22 cells, which may be related to ferroptosis reduction and Nrf2/HO1 signaling pathway activation.

## Materials and methods

### Materials

Salidroside (Macklin, S817419, purity > 98%), Dimethyl sulfoxide (DMSO, sigma-Aldrich, D2650), Ferrostatin-1(Fer-1, Topscience, 347174-05-4) was dissolved in DMSO and freshly diluted to the final concentration (0.05% DMSO) in the study, Glutamate(Glu, sigma-Aldrich, G1626), Amyloid β Protein Fragment 1–42(Aβ_1− 42_, sigma-Aldrich, G1626), Cell Counting Kit-8 assay kit (CCK-8, bimake, B34304, USA), Lactate Dehydrogenase (LDH) detection kit (Best Bio, China, BB-4860-500T), Reactive Oxygen Species (ROS) assay kit (Nanjing jiancheng, E004-1-1), JC-1(Solarbio, J8030), C11 BODIPY 581/591(GLPBIO, 217075-36-0), FerroOrange Cell Ferrous Ion Fluorescence Probe (Dojindo, Japan, F374), Lipid Peroxidation MDA Assay Kit (Beyotime, S0131S), Total Superoxide Dismutase Assay Kit with WST-8 (Beyotime, S0101S), GSH and GSSG Assay Kit (Beyotime, S0053), LipoInsect™ Transfection Reagent (Beyotime, C0526-1.5mL), Opti-MEM™ I Reduced Serum Medium (Gibco, 31985070), Alexa Fluor 488-labeled Goat Anti-Rabbit IgG(H + L) (Beyotime, A0423), GPX4 Antibody (Affinity, DF6701), SLC7A11 Polyclonal Antibody (Proteintech, 26864-1-AP), HO1 Antibody (Affinity, AF5393), Nrf2 Polyclonal Antibody (Proteintech, 16396-1-AP), PCNA Polyclonal Antibody (Proteintech, 10205-2-AP), β-Actin (D6A8) Rabbit mAb (Cell Signaling Technology, #8457), HRP-conjugated Affinipure Goat Anti-Rabbit IgG(H + L) (Proteintech, SA00001-2).

### Cell culture

The immortalized mouse hippocampal cell line HT22 were cultured in DMEM supplemented with 10% fetal bovine serum (FBS, Gibco, A31608-02), 100 U/mL penicillin, and 100 µg/mL streptomycin (Gibco, 10378016) in a 37 ℃ containing 5% CO_2_ humidified incubator. Cells were dissociated at a 1:3 ratio by 2.5% trypsin when growth reached 90-95% confluence.

### Cell viability

**Fig. 1:**
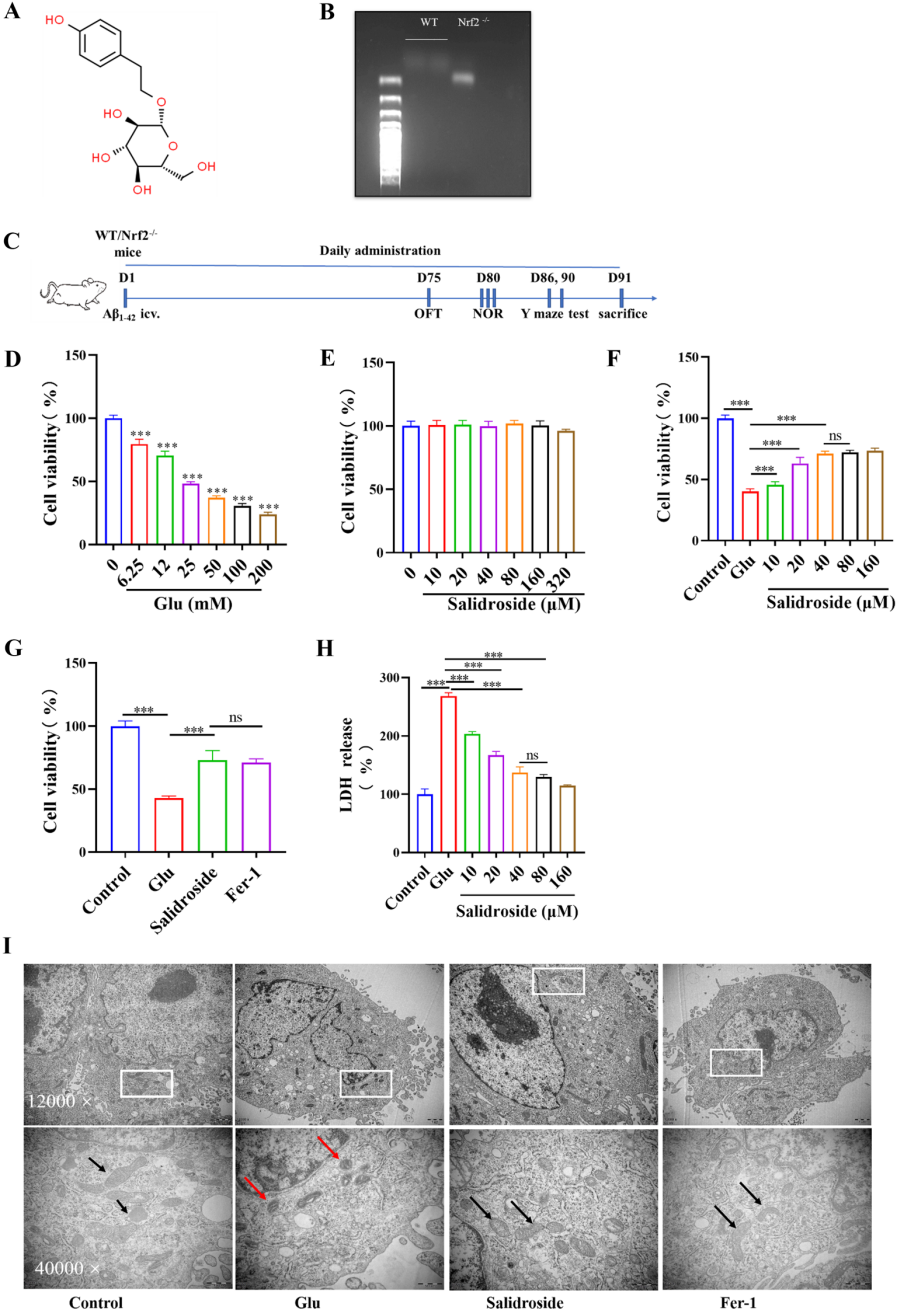
Salidroside increases the cell viability in Glu-injured HT22 cells. **A** Chemical structure of salidroside. **B** Mouse genotypes were confirmed by PCR of tail DNA. **C** The animal experimental process. **D** Effects of different concentrations Glu on the HT22 cells viability. ***P < 0.001 when compared with the control group (Glu = 0 mM). **E** Effects of different concentrations salidroside on the HT22 cells viability. **F** Effects of different concentrations salidroside on the viability of Glu-injured HT22 cells. *P < 0.05, **P < 0.01, ***P < 0.001, “ns” means no significant difference (*P* >  0.05). **G** Effects of salidroside and Fer-1 on the viability of Glu-injured HT22 cells. *P < 0.05, **P < 0.01, ***P < 0.001. **H** The CCK-8 assay results were confirmed by the cell LDH assay. *P < 0.05, **P < 0.01, ***P < 0.001, “ns” means no significant difference (*P* >  0.05). **I** TEM showed the ultrastructural changes of mitochondria in each group. The Glu group showed that mitochondria became smaller and the density of bilayer membrane increased. Salidroside and fer-1 could improve this change

The HT22 cells were seeded in 96-well plates at a density of 5 × 10^3^ cells/well. The optimal concentration of salidroside (Macklin, Lot: C10739039) was explored with a cell viability assay. The chemical structure of salidroside is shown in Fig. 1A. The effects of salidroside against Glu toxicity were assessed. In this study, cells were pretreated with salidroside for 24 h and then exposed to Glu (25 mM) in the presence of salidroside (cotreatment) for another 24 h. Ferrostain-1 (5 µM, Fer-1, Topscience, 347174-05-4) was used as a positive control. A Cell Counting Kit-8 (CCK-8, Bimake, B34304, USA) was used to assess the cells, and the OD_450_ was measured. The cell survival rates were calculated as follows: survival rate (%) = (experimental absorbance value/control absorbance value) × 100%.

### LDH release assay

Cytoplasmic enzymes, including lactate dehydrogenase (LDH), are released into the supernatant after cell death. The release of LDH was regarded as an indicator of cell membrane integrity. In this study, an LDH Cytotoxicity Assay Kit (Best Bio, China, BB-4860-500T) was used to detect LDH release according to the manufacturer’s protocol.

### Observation of cell ultrastructure by transmission electron microscopy

HT22 cells were seeded at a density of 1 × 10^6^ cells/well in 6-well plates. The cells were incubated with Glu, salidroside, and Fer-1 as mentioned above. Cell samples were fixed with 2.5% glutaraldehyde for more than 24 h, rinsed with PBS, fixed with 1% osmium acid, and then rinsed with PBS. Samples were dehydrated and embedded. The osmotic-treated samples were placed in 0.5 mL Eppendorf tubes, embedded, and heated at 70 °C overnight. Samples were cut into ultrathin sections and stained with 2% uranyl acetate for 20 min and 0.04% lead citrate for 10 min, and the ultrastructure was observed by transmission electron microscopy (TEM) (Hitachi H-7500, Japan).

### Analysis of intracellular Fe^2+^ by FerroOrange staining

To assess intracellular Fe^2+^, HT22 cells were seeded at a density of 1 × 10^6^ cells/well in 6-well plates. The cells were incubated with Glu, salidroside, and Fer-1 as mentioned above. For imaging of intracellular Fe^2+^, HT22 cells were stained with serum-free phenol red-free DMEM (Thermo Fisher Scientific, Cat# 21063029) containing 1 µM FerroOrange (Dojindo, Japan, F374) for 30 min at 37 °C in a CO_2_ incubator. Fluorescence measurements were conducted using fluorescence digital microscopy (Olympus IX 53, Tokyo, Japan). Fluorescence intensity was quantified using ImageJ.

### Analysis of reactive oxygen species, mitochondrial membrane potential, and lipid peroxidation by fluorescence microscopy

HT22 cells were seeded at a density of 1 × 10^6^ cells/well in 6-well plates and incubated with Glu, salidroside, and Fer-1 as mentioned above. 2,7-Dichlorofluorescin diacetate (DCFH-DA) (Nanjing Jiancheng, E004-1-1), JC-1 (Solarbio, J8030), and C11 BODIPY 581/591 (GLPBIO, 217075-36-0) were diluted to 10 µM, 10 µg/mL and 1 µM, respectively, in serum-free medium and added to the wells, and the mixture was incubated in the dark at 37 °C for 20 min. After 2 washes with PBS, the cells were observed with a fluorescence microscope. For ROS analysis, green fluorescence intensity was measured at 530 nm. For the mitochondrial membrane potential (MMP) analysis, the ratio of the JC-1 monomer of the dye (green fluorescence at 530 nm) to the JC-1 polymer (red fluorescence at 590 nm) of the dye was calculated. For the lipid peroxidation analysis, the ratio of the oxidized form (green fluorescence at 530 nm) to the normal form (red fluorescence at 590 nm) of the dye was calculated.

### Quantitative analysis of ROS, MMP, and lipid peroxidation by flow cytometry

HT22 cells were seeded at a density of 5 × 10^6^ cells/well in 6-well plates. Cells were treated and stained as above. Next, the culture medium was aspirated, and the cells were collected and centrifuged for 3 cycles of 5 min at 1500 rpm. After the supernatant was removed, the cells were resuspended with PBS and then analyzed with a CytoFLEX flow cytometer (Becton Dickinson, USA). Data are documented as the percentage of fluorescence intensity.

### Measurement of MDA, SOD, and GSH/GSSG

Malondialdehyde (MDA) is a natural product of lipid oxidation. When oxidative stress occurs in animal or plant cells, many adverse reactions to lipid oxidation occur. Some fatty acids gradually decompose into a series of complex compounds after oxidation, including MDA. At this time, lipids can be assessed by measuring the level of MDA. Therefore, the assessment of MDA is widely used as an indicator of lipid oxidation. The MDA content was measured according to the manufacturer’s protocol by a Lipid Peroxidation MDA Assay Kit (Beyotime, S0131S). Superoxide dismutase (SOD) can catalyze the disproportionation of superoxide anions to produce hydrogen peroxide (H_2_O_2_) and oxygen (O_2_), which are important antioxidant enzymes in organisms. The SOD content in cells was quantified according to the manufacturer’s recommended protocol by a Total Superoxide Dismutase Assay Kit with WST-8 (Beyotime, S0101S). Glutathione includes reduced glutathione (GSH) and oxidized glutathione disulfide (GSSG). Reduced glutathione is a key antioxidant in animal cells. The concentrations of GSH and GSSG in cells were measured according to the manufacturer’s recommended protocol by a GSH and GSSG Assay Kit (Beyotime, S0053).

### Western blot analysis

HT22 cells were treated as described above. Whole-cell protein, nuclear and cytoplasmic protein were extracted by a whole-cell lysis assay (Key GEN Bio TECH, China, KGP250) and nuclear and cytoplasmic protein extraction kit (Key GEN Bio TECH, China, KGP150). For protein quantification, the bicinchoninic acid (BCA) protein quantitation assay (Key GEN Bio TECH, China, KGPBCA) was used according to the manufacturer’s instructions. The extracted protein was added to a 5× loading buffer (Pythonbio, China, AAPR39-1) and boiled. Equal amounts of the protein were separated on 12% sodium dodecyl sulfate (SDS)-polyacrylamide gels, followed by transfer to polyvinylidene fluoride (PVDF) membranes (Millipore, USA, ISEQ00010). The membranes were blocked with 5% skim milk in Tris-buffered saline containing 0.01% Tween 20 (TBST) at room temperature for 1 h, followed by a reaction with primary antibodies against β-actin, SLC7A11, GPX4, Nrf2, and HO1. The membranes were washed 3 times with TBST for 10 min and incubated with horseradish peroxidase (HRP)-conjugated goat anti-rabbit antibody. After the membranes were washed 3 times with TBST for 10 min, the target protein bands were detected using the FluorChem^®^ M MultiFluor system (Cell Biosciences, CA, USA). β-Actin was measured as a loading control, and the densities of the protein bands were measured by using Image J.

### Anti-Nrf2 immunofluorescence in HT22 cells

Nuclear translocation of Nrf2 was analyzed by immunofluorescence. HT22 cells were treated as described above. The cells were fixed with 4% formaldehyde for 30 min at room temperature and then permeabilized in 0.4% Triton X-100 in PBS for 15 min. Then, the cells were blocked in 5% BSA for 1 h at room temperature and incubated for 12 h at 4 °C with the anti-Nrf2 antibody. After being washed twice with PBS, the cells were incubated for 1 h with Alexa Fluor 488-labeled goat anti-rabbit IgG (H + L) at room temperature. Images were acquired with an Aser scanning confocal microscope (Carl Zeiss, LSM800 With Airyscan, Germany).

### siRNA Nrf2 transfection

HT22 cells were seeded at a density of 3 × 10^4^ cells/well into laser confocal culture dishes. When they reached approximately 70–80% confluence, the HT22 cells were transfected with Nrf2 siRNA (siG2006160258184354, si-m-Nrf2-001, RIBOBIO, Guangzhou) for 24 h using LIP 6000 Transfection Reagent (Beyotime, China, C0526). The efficiency of silencing was confirmed by qRT PCR after 24 h of transfection.

### Animals experiment

B6.129 × 1-*Nfe2l2*^tm1Ywk^/J(*Nrf2*^−/−^mice, 017009)and wild-type C57BL/6 were originally from the Jackson Laboratory. Grouping and administration were started when weighing approximately 28-33 g. All mice were housed in a laboratory environment with free access to adequate food and water under a 12 h/12 h light/dark cycle at 22 ± 1 °C and 55 ± 5% humidity. All procedures conformed to the protocols of the Animal Welfare Commission and Ethical Committee of Southern Medical University. The Nrf2^−/−^ mice were identified by genotyping as shown in Fig. 1B. WT and Nrf2^−/−^ mice were randomly assigned to 3 × 2 groups (3 groups for WT mice and 3 groups for Nrf2^−/−^ mice) as follows: a sham group, sham + Aβ_1−42_ group, and Salidroside + Aβ_1−42_ group. The animal experimental process is shown in Fig. 1C.

### Animals, stereotaxic injections, and treatmentwith salidroside

Surgical procedures and intracerebroventricular injection (i.c.v.) of Aβ_1−42_ (222 µM) into the right hemisphere were performed. In brief, 2 µL Aβ_1−42_ was injected at the following coordinates [30]: − 0.1 mm anteroposterior (AP), − 0.75 mm mediolateral (ML), and − 1.8 mm dorsoventral (DV) relative to bregma. WT mice and Nrf2^−/−^ mice (Salidroside + Aβ_1−42_ group) were treated with salidroside at a dosage of 50 mg/kg/day for 75 days [22]. Control mice were given saline following the same routine. The different groups of mice (n = 6) were subjected to the novel object recognition test, Y maze, and open field test. Transmission electron microscopy was performed to observe mitochondrial ultrastructure in the hippocampal CA1 region. Immunohistochemistry was performed to detect the expression of GPX4 in the hippocampus and cortex. The expression of SLC7A11, GPX4, Nrf2, and HO1 in the hippocampus were investigated by Western blot.

### Novel object recognition test (NORT)

The standard and modified novel objective recognition tests were performed as described previously [31]. Smart V3.0 Panlab software was used for analysis. The exploration time of the object was defined as the time the mice spent with its nose within 2 cm of the object. Discrimination index (%) = [(novel object exploration-familiar object exploration time)/(novel exploration time + familiar object exploration)] ×100%.

### Y-maze test

The Y-maze test was conducted as previously reported [32], and the alternation percentage was calculated as follows: Alternation percentage (%) = (number of alternations)/(total number of arms entires-2) ×100%. The novel arm Y-maze test method was performed as described [22], and the time spent in the novel arm was calculated as follows: The time spent in the novel arm (%) = (time spent in the novel arms/total time spent in all arms) ×100%.

### Open field test (OFT)

The OFT measured general locomotor activity. Spontaneous activity was measured in a clear plastic box (40 × 40 × 40 cm) under a camera. Each mouse was placed in the center of the open field arena and allowed to move freely for 5 min. The total distance traveled was analyzed.

### Transmission electron microscopic observation

After the behavioral test, the hippocampus was collected and placed in 2.5% glutaraldehyde fixative. Tissues were cut into pieces of 0.5–1 mm^3^ and placed in 2.5% glutaraldehyde fixative. Tissues were fixed for 1 h at room temperature and then overnight at 4 °C. Glutaraldehyde was replaced with PBS, and the tissues were stored in an EP tube at 4 °C. Sections were photographed using a light microscope and then serially cut into 1–2 μm semithin sections. Selected semithin sections were sliced into serial ultrathin sections with a silver-gray interference color, corresponding to a thickness of 50–70 nm. Ultrathin sections were collected on formvar-coated, single-slot grids and stained with uranyl acetate and lead citrate. Neurons in the hippocampal CA1 region were observed and photographed by TEM (Hitachi H-7500, Japan).

### Immunohistochemistry

Mouse brain paraffin-embedded sections from each group were dewaxed and placed in EDTA buffer (pH 9.0) for antigen repair. The sections were blocked with 3% (v/v) H_2_O_2_ for 10 min at room temperature and then incubated in 1% Triton X-100 for 10 min at room temperature. After blocking with 10% (w/v) BSA in PBS for 15 min, the sections were incubated with an antibody against GPX4 overnight at 4 °C. Finally, the sections were incubated with HRP-conjugated secondary antibody. The color was developed with DAB chromogenic, and the sections were counterstained with hematoxy*lin.* The hippocampus and cortex were observed at 200× magnification using an automatic digital pathology slice scanner (KFBOI KF-PRO-005, China) and used to scan the immunohistochemically stained sections.

### Statistical analysis

Quantitative data are represented as the mean ± standard deviation (SD). Statistical analyses were completed using the Statistical Package for Social Science (SPSS) program, version 25 (SPSS Inc., USA). Comparisons among groups were analyzed using one-way analysis of variance (ANOVA) with Tukey’s posthoc multiple comparisons test. The results were considered statistically significant when *P* < 0.05. Data were tabulated and plotted using GraphPad Prism, version 8 (USA).

## Results

### Salidroside increased the cell viability in glu-injured HT22 cells

Glu toxicity in HT22 cells was explored with a CCK-8 assay. The cell viability decreased to 48.15%±1.58% (Fig. 1D) when cells were exposed to 25 mM of Glu for 24 h. The concentration is consistent with previous studies [33–35] and was chosen in the following experiments. No significant differences were observed in the salidroside groups and the negative control (Fig. 1E) after pretreatment with different concentrations of salidroside (0–320 µM) for 24 h. The protective effect of salidroside against Glu-injured cell death was investigated. The protective effect was highest at 40 µM of salidroside (71.15%±1.84% cell viability, *P* < 0.001 when compared to the Glu group; Fig. 1F, G). Therefore, the salidroside concentration was used in the following experiments. The CCK-8 assay results were confirmed by the LDH release assay (Fig. 1H). These findings showed that salidroside has a potential neuroprotective effect against Glu-injured cytotoxicity in HT22 cells.

### Salidroside increased the mitochondrial membrane potential and decreased the levels of intracellular Fe^2+^

**Fig. 2:**
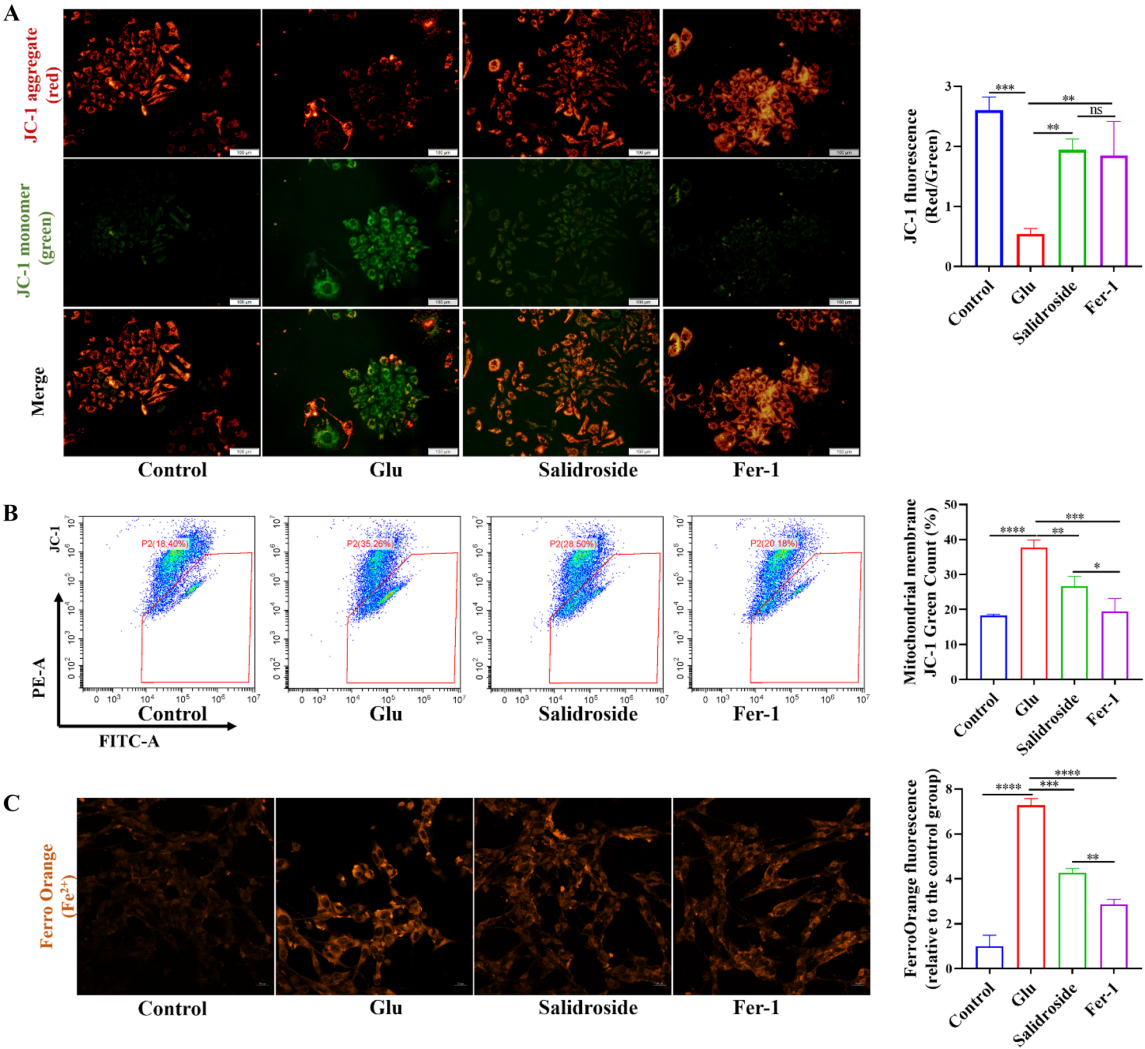
Salidroside improved the MMP and decreased the levels of intracellular Fe^2+^. **A** JC-1 staining indicated that salidroside can increase the MMP of the HT22 cells. **B** Flow cytometric detection suggested that salidroside increased the MMP of the HT22 cells. **C** Salidroside and Fer-1 decreased the content of cell Fe^2+^. *P < 0.05, **P < 0.01, ***P < 0.001, ****P < 0.0001

Alterations in mitochondrial ultrastructure were observed by TEM. After salidroside and Fer-1 treatment, mitochondria had regular morphology and less vacuolation, while in the Glu group, the mitochondrial membrane density increased and mitochondrial cristae disappeared (Fig. 1I). The critical indicator of mitochondrial function is MMP. In the study, MMP was detected by JC-1 probe staining. Red fluorescence is produced when the MMP is high, and JC-1 aggregates to form a polymer. However, when the MMP is low, JC-1 is a monomer and can produce green fluorescence. The results indicated that the MMP of the salidroside group was higher than that in the Glu group (Fig. 2A). The results were confirmed by flow cytometric detection (Fig. 2B). Divalent iron can produce excess ROS through the Fenton reaction, increase the activity of ester oxygenase, catalyze the highly expressed unsaturated fatty acids on the cell membrane, produce lipid peroxidation and induce cell death [14]. A FerroOrange probe, which can react specifically with Fe^2+^ in living cells, was used to observe the accumulation of Fe^2+^ in living cells. A previous study also confirmed that the increase of ferrous ions was found in the treatment of high concentrations of Glu [36]. The results showed that salidroside and Fer-1 decreased the content of cellular Fe^2+^ compared with that in the Glu group (Fig. 2C).

### Salidroside decreased the accumulation of oxidation products in HT22 cells

**Fig. 3:**
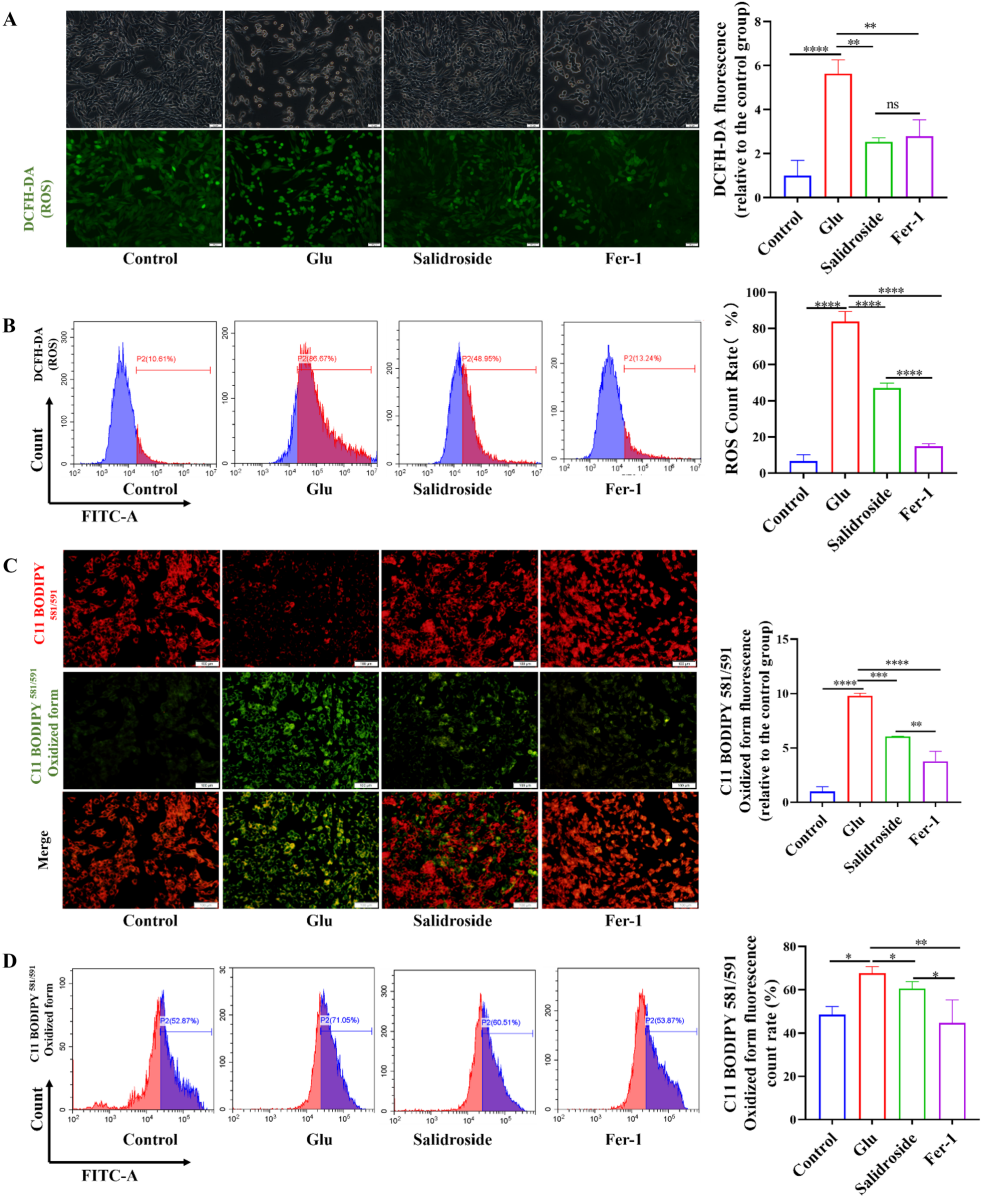
Salidroside decreased the accumulation of oxidation products of HT22 cells. **A, B** Salidroside decreased production of intracellular ROS. **C, D** Salidroside decreased the accumulation of lipid peroxidation. *P < 0.05, **P < 0.01, ***P < 0.001, ****P < 0.0001

**Fig. 4:**
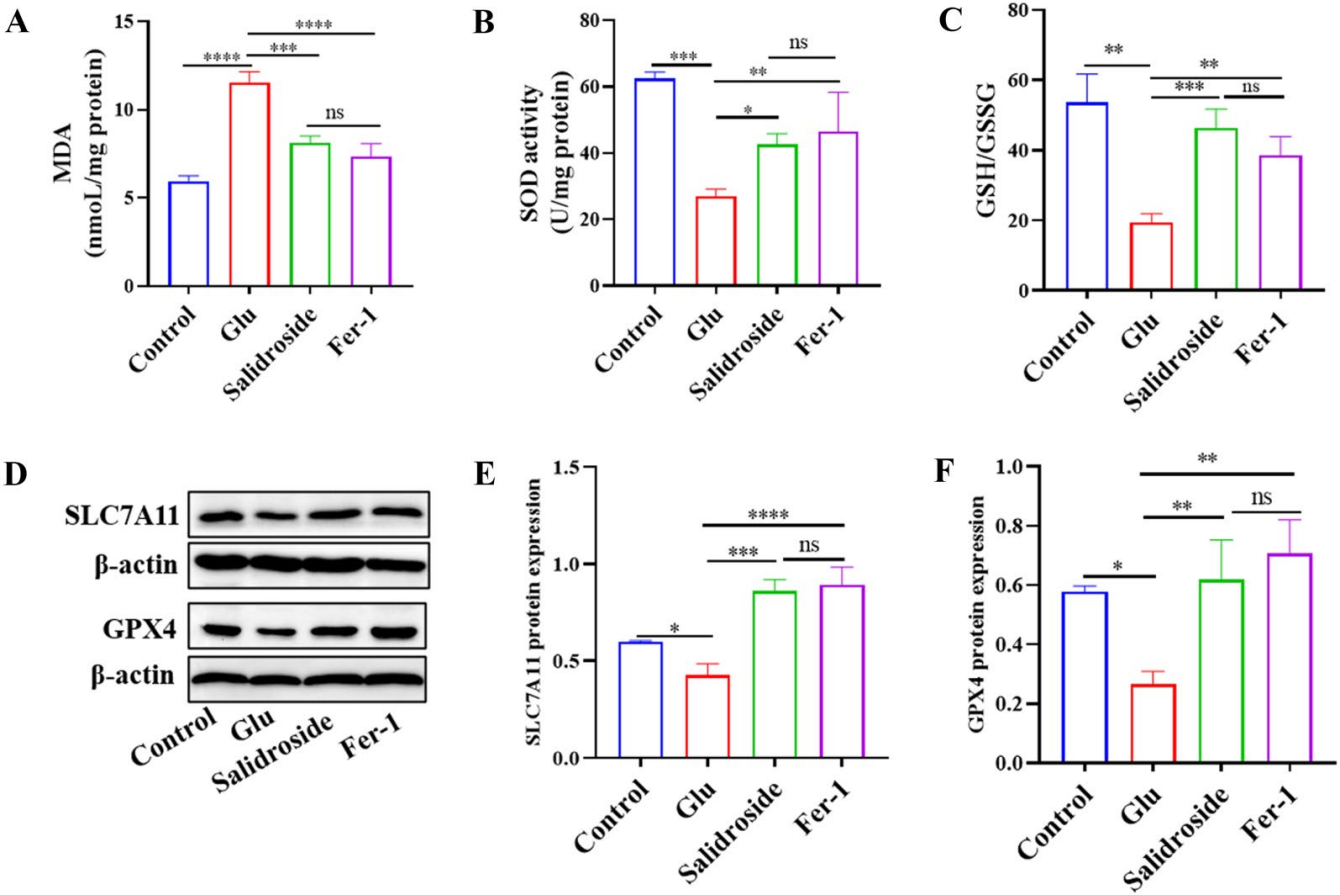
Salidroside reduced the oxidative stress of Glu-injured HT22 cells and increased the protein expression of SLC7A11 and GPX4. **A** Salidroside decreased the content of MDA in Glu-injured HT22 cells when compared with the Glu group. **B, C** The content of SOD and the ratio of GSH/GSSG in the salidroside group were increased when compared with the Glu group. **D–F** The expression of GPX4 and SLC7A11 in the salidroside group were increased when compared with the Glu group. *P < 0.05, **P < 0.01, ***P < 0.001, “ns” means no significant difference (*P* > 0.05)

A DCFH-DA probe was used to assess intracellular ROS. Compared with the control group, treatments with salidroside and Fer-1 significantly decreased intracellular ROS accumulation (Fig. 3A). The results were confirmed by flow cytometric detection (Fig. 3B). C11 BODIPY 581/591 is a lipid-soluble ratiometric fluorescent indicator of lipid oxidation. Upon oxidation, its excitation maximum shifts from 581 to 500 nm, and the emission maximum shifts from 591 to 510 nm. C11 BODIPY ^581/591^ has been used in biochemical assays and live-cell applications. C11 BODIPY ^581/591^ was used to detect lipid peroxidation, and the results showed that the lipid peroxidation level of the salidroside group was lower than that of the Glu group (Fig. 3C). The flow cytometry results also corresponded to these results (Fig. 3D). The MDA, GSH/GSSG, and SOD contents were measured in HT22 cells. The results showed that the SOD content and the GSH/GSSG ratio in the salidroside group were increased when compared with the Glu group. The MDA content in the salidroside group was decreased when compared with the Glu group. (Fig. 4A–C).

### Salidroside inhibited ferroptosis by increasing expression of GPX4 and SLC7A11 in glu-injured HT22 cells

The expression of GPX4 and SLC7A11 were detected by Western blot. We found that the expression of GPX4 and SLC7A11 in the salidroside group was higher than that in the Glu group (Fig. 4D–F). Salidroside inhibited ferroptosis in Glu-injured HT22 cells by upregulating the expression of GPX4 and SLC7A11.

### Salidroside inhibited ferroptosis by activating the Nrf2/HO1 pathways in glu-injured HT22 cells

**Fig. 5:**
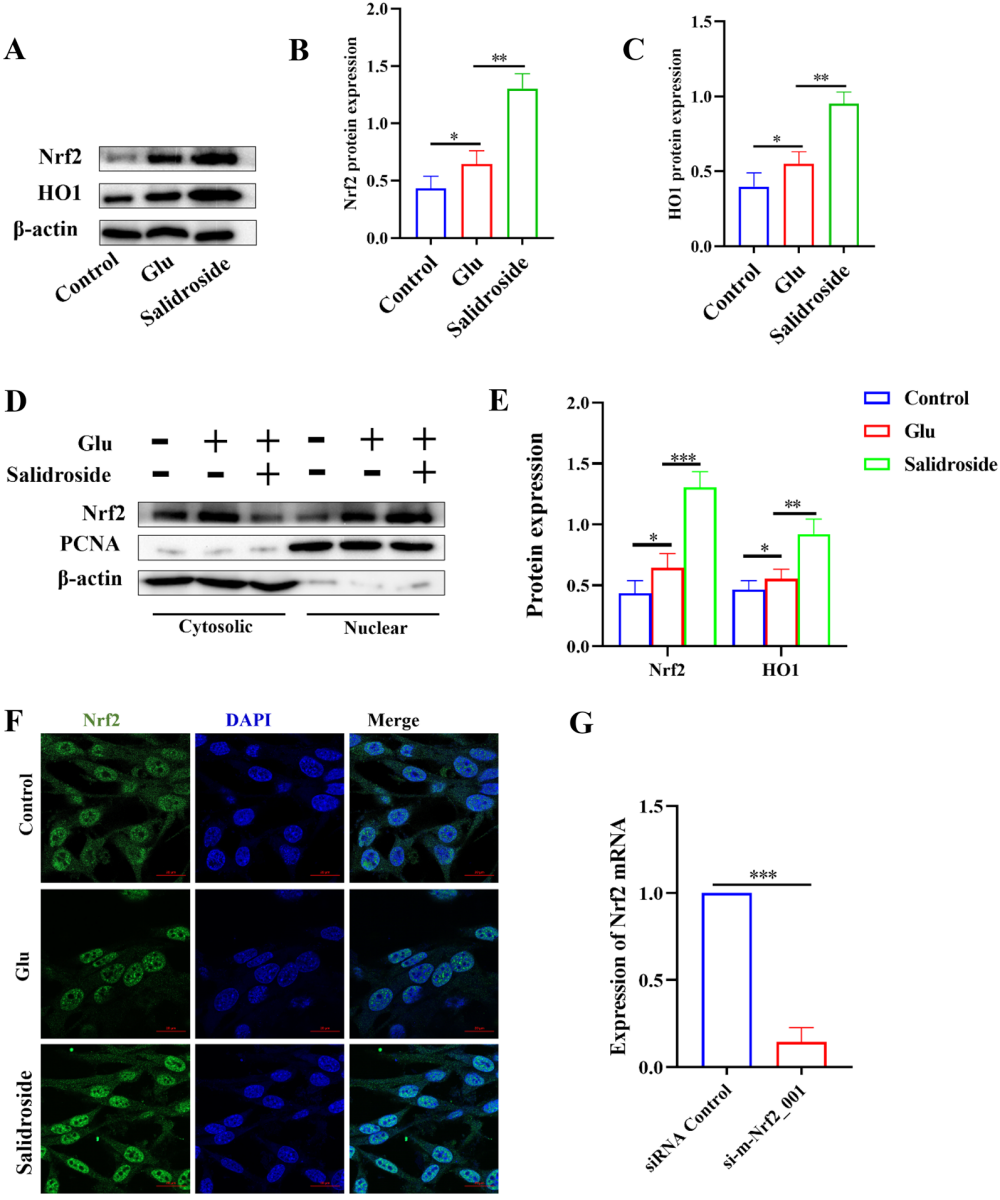
Salidroside inhibited ferroptosis by activating the Nrf2/HO1 pathway in Glu-injured HT22 cells. **A-C** The expression of Nrf2 and HO1 in the salidroside group were increased when compared with the Glu group. **D, E** Western blot showed that salidroside increased nuclear Nrf2 expression. **F** Immunofluorescence showed that salidroside increases the Nrf2 nuclear translocation in Glu-injured HT22 cells. **G** Nrf2 siRNA transfection efficiency on the HT22 cells. *P < 0.05, **P < 0.01, ***P < 0.001

Western blot results indicated that Glu increased the levels of Nrf2, which is consistent with the findings of Park, H. et al. [37] reported. The same trend of Nrf2 and HO1 expression suggests that excess cytoplasmic Nrf2 is transferred to the nucleus and increases HO1 expression. Co-treatment of salidroside with Glu significantly increased Nrf2/HO1 levels compared to the Glu group (Fig. 5A–C), suggesting that the activation of the Nrf2/HO1 signaling pathway may be one of the possible mechanisms of salidroside. The expression of cytoplasmic and nuclear Nrf2 were examined and the results showed that the salidroside increases the nuclear translocation of Nrf2 (Fig. 5D, E). Correspondingly, immunofluorescence showed that salidroside increases the nuclear transport of Nrf2 (Fig. 5F).

**Fig. 6:**
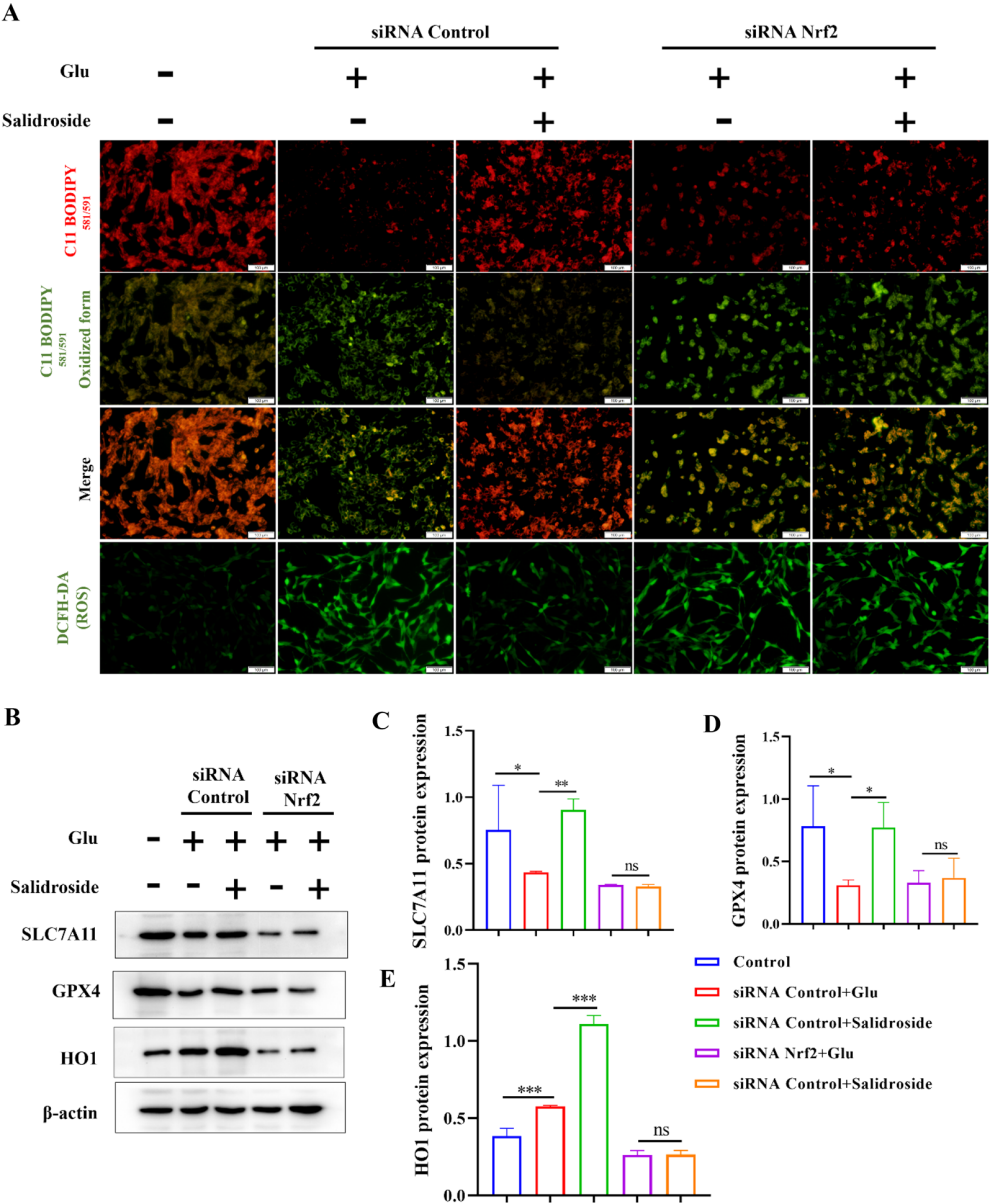
Salidroside inhibited ferroptosis by activating the Nrf2/HO1 pathways on Glu-injured HT22 cells. **A** The effects of salidroside decrease the level of intracellular ROS and lipid peroxidation was attenuated on siRNA Nrf2 transfected HT22 cells. **B–E** The effects of salidroside increased the level of GPX4, SLC7A11 and HO1were weakened after siRNA Nrf2 transfection. *P < 0.05, **P < 0.01, ***P < 0.001, “ns” means no significant difference (*P* > 0.05)

To investigate the role of Nrf2 in ferroptosis in HT22 cells, Nrf2 siRNA was transfected into HT22 cells (Fig. 5G). DCFH-DA and the C11 BODIPY 581/591 probe were used to detect the levels of intracellular ROS and lipid peroxidation. The effects of salidroside decreased intracellular ROS, and lipid peroxidation was inhibited after siRNA Nrf2 transfection. (Fig. 6A). Western blot analysis showed that salidroside increases the expression of GPX4, SLC7A11, and HO1 in Glu-injured HT22 cells, which was weakened after siRNA Nrf2 transfection (Fig. 6B– E). The data suggested that salidroside inhibited ferroptosis by activating the Nrf2/HO1 pathways in HT22 cells.

### **Cognitive impairment induced by Aβ**_**1−42**_**was mitigated by salidroside in WT mice but not in Nrf2**^−/−^**mice**

**Fig. 7:**
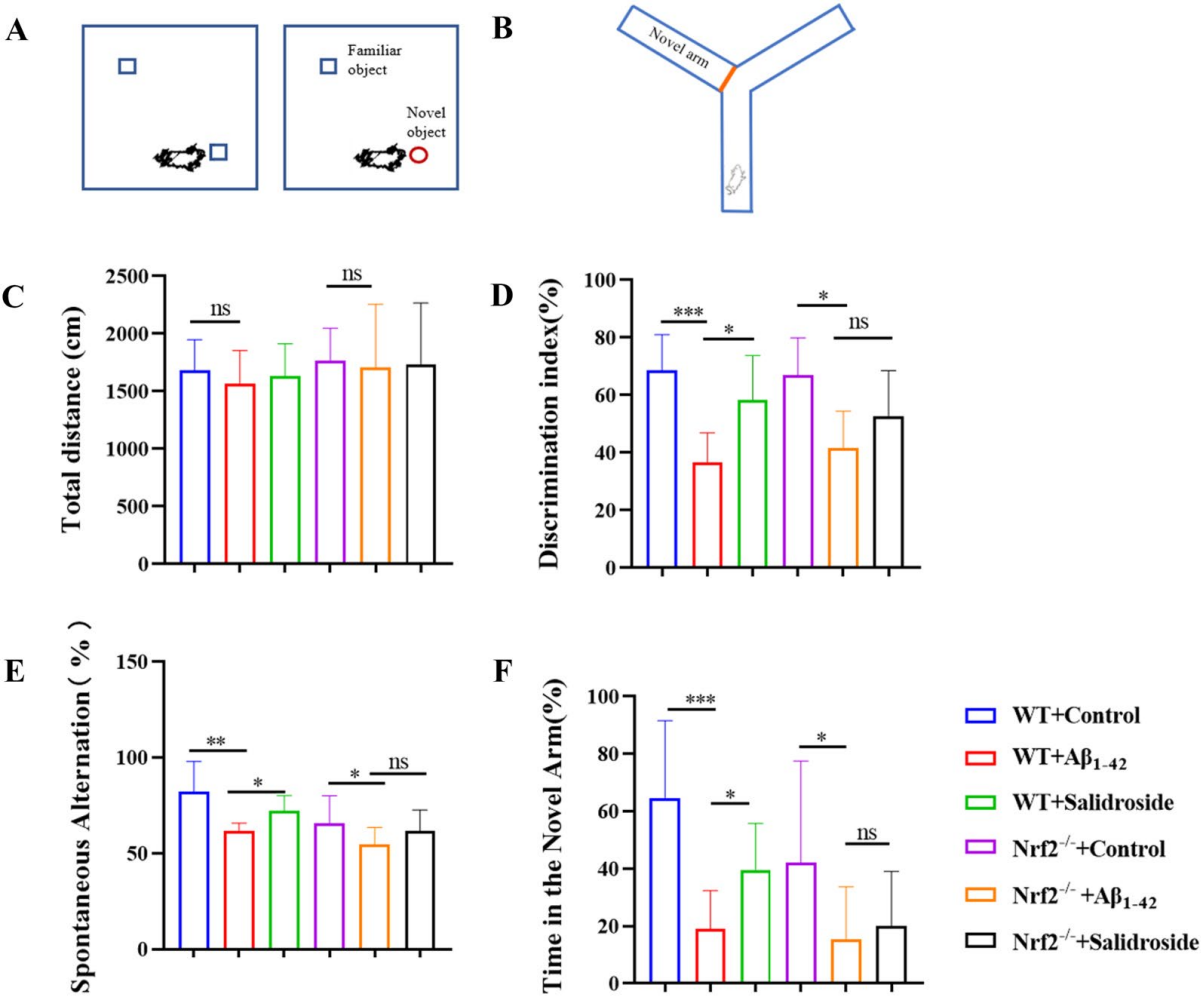
Cognitive impairment induced by Aβ_1−42_ was mitigated by salidroside in WT mice, but not in Nrf2^−/−^mice. **A** Schematic diagram of NORT. **B** Schematic diagram of Y maze. **C** OFT results showed no significant difference was observed in the total distance between the Aβ_1−42_ group and salidroside treatment group in Nrf2^−/−^ mice and WT mice. **D** There was no preference for the novel object in the Aβ_1−42_-Nrf2^−/−^ mice after salidroside treatment, whereas those Aβ_1−42_-WT mice administered with salidroside performed comparable to the WT mice. **E** Spontaneous alternation (%) showed Aβ_1−42_ -treated mice decreased alternations while salidroside reversed this impairment in WT mice but not Nrf2^−/−^ mice. **F** Time in the novel arm (%) was reduced after Aβ_1−42_ treatment, the decrease after salidroside was reversed in WT mice but not Nrf2^−/−^ mice. *P < 0.05, **P < 0.01, ***P < 0.001, “ns” means no significant difference (*P* > 0.05)

We established an AD mice induced by Aβ_1−42_ and evaluated cognitive function to assess the therapeutic effects of salidroside on Aβ_1−42_-induced cognitive impairment. The OFT, NORT, and Y mazes were performed to assess the cognitive function of the AD mice (Fig. 7A, B). No significant difference was observed in the total distance between the Aβ_1−42_ and salidroside treatment groups in Nrf2^−/−^ mice and WT mice (Fig. 7C). The spatial and working memory of the mice was evaluated by the NORT. There was no preference for the novel object in the Aβ_1−42_-Nrf2^−/−^mice after salidroside treatment, whereas those Aβ_1−42_-WT mice administered with salidroside performed comparably to the WT mice (Fig. 7D). Spatial memory was assessed by the Y-maze test, and spontaneous alternation showed that Aβ_1−42_-treated mice exhibited decreased alternations, while salidroside reversed this impairment in WT mice but not Nrf2^−/−^ mice (Fig. 7E). Spatial recognition tests showed that the novel arm exploration time was reduced after Aβ_1−42_ treatment, indicating impaired spatial memory. The cognitive impairment was reversed in WT mice but not in Nrf2^−/−^mice after salidroside treatment (Fig. 7F).

### **Salidroside alleviated Aβ**_**1−42**_**-induced ferroptosis in the hippocampus in WT mice, but not in the Nrf2**^**−/−**^**mice**

**Fig. 8:**
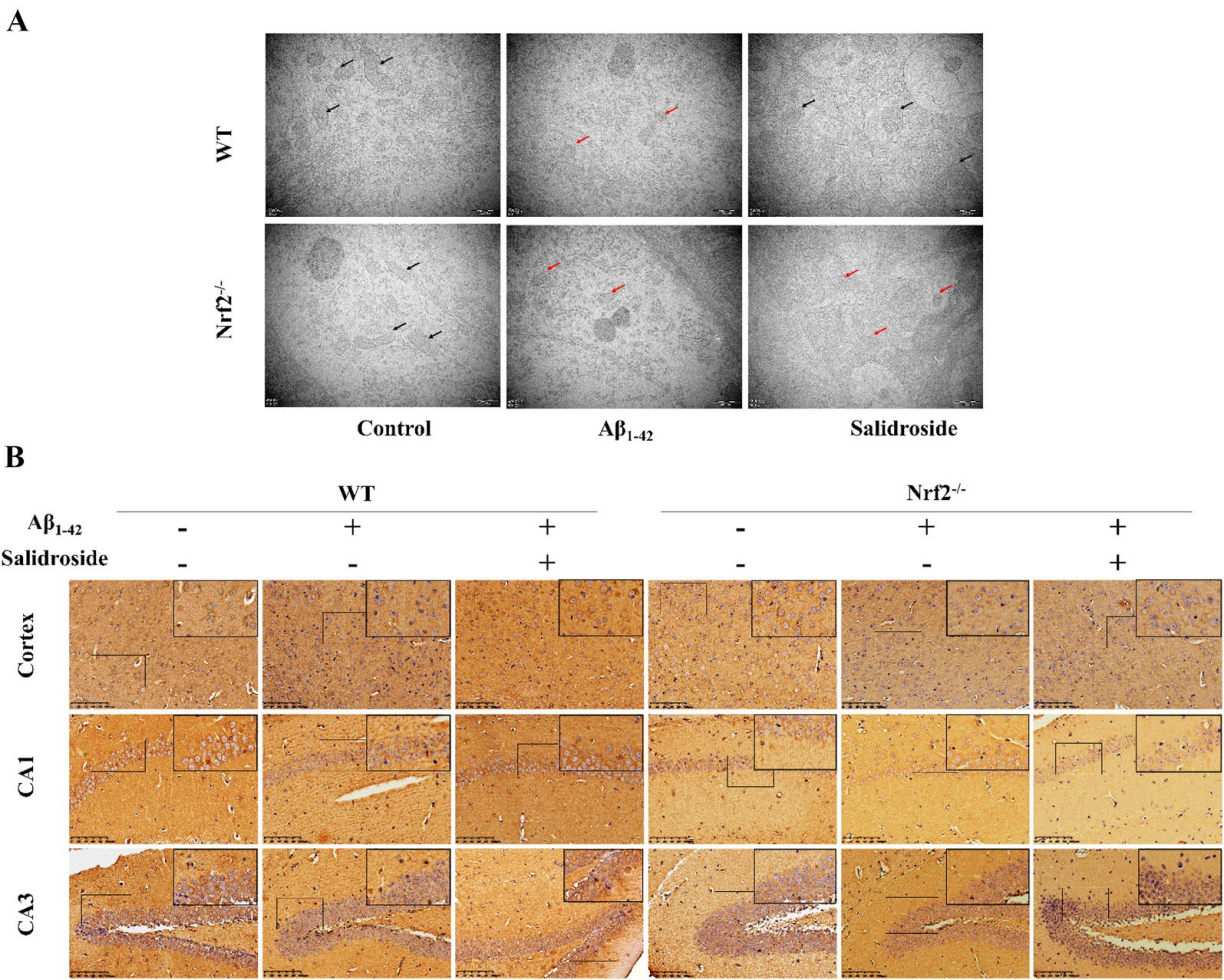
Salidroside regulated neurons mitochondrial ultrastructural changes and GPX4 expression in WT mice, but not in Nrf2^−/−^mice. **A** Ultrastructural changes in neuronal mitochondria in the hippocampal CA1 region. The mitochondria observed in the Aβ_1−42_ group were smaller than those in the control group, and the cristae of mitochondria in the Aβ_1−42_ group were decreased. Treatment with salidroside attenuated these morphological changes in WT mice but not in Nrf2^−/−^ mice. Scale bars: 500 nm. **B** Immunohistochemistry showed that salidroside increased the expression of GPX4 in hippocampus and cortex in Aβ_1−42_ induced WT mice, but this change was not observed in Nrf2 ^−/−^ mice. Scale bar: 100 μm

TEM was performed to observe the ultrastructure of neuron mitochondria in the hippocampal CA1 region. The mitochondria in the Aβ_1−42_ group were smaller than in the control group, and the cristae of mitochondria in the Aβ_1−42_ group were decreased. Treatment with salidroside attenuated these morphological changes induced by Aβ_1−42_ in WT mice but not in Nrf2^−/−^ mice (Fig. 8A).

### **Salidroside regulated the expression of ferroptosis-related proteins in the WT mice, but not in the Nrf2**^**−/−**^**mice**

**Fig. 9:**
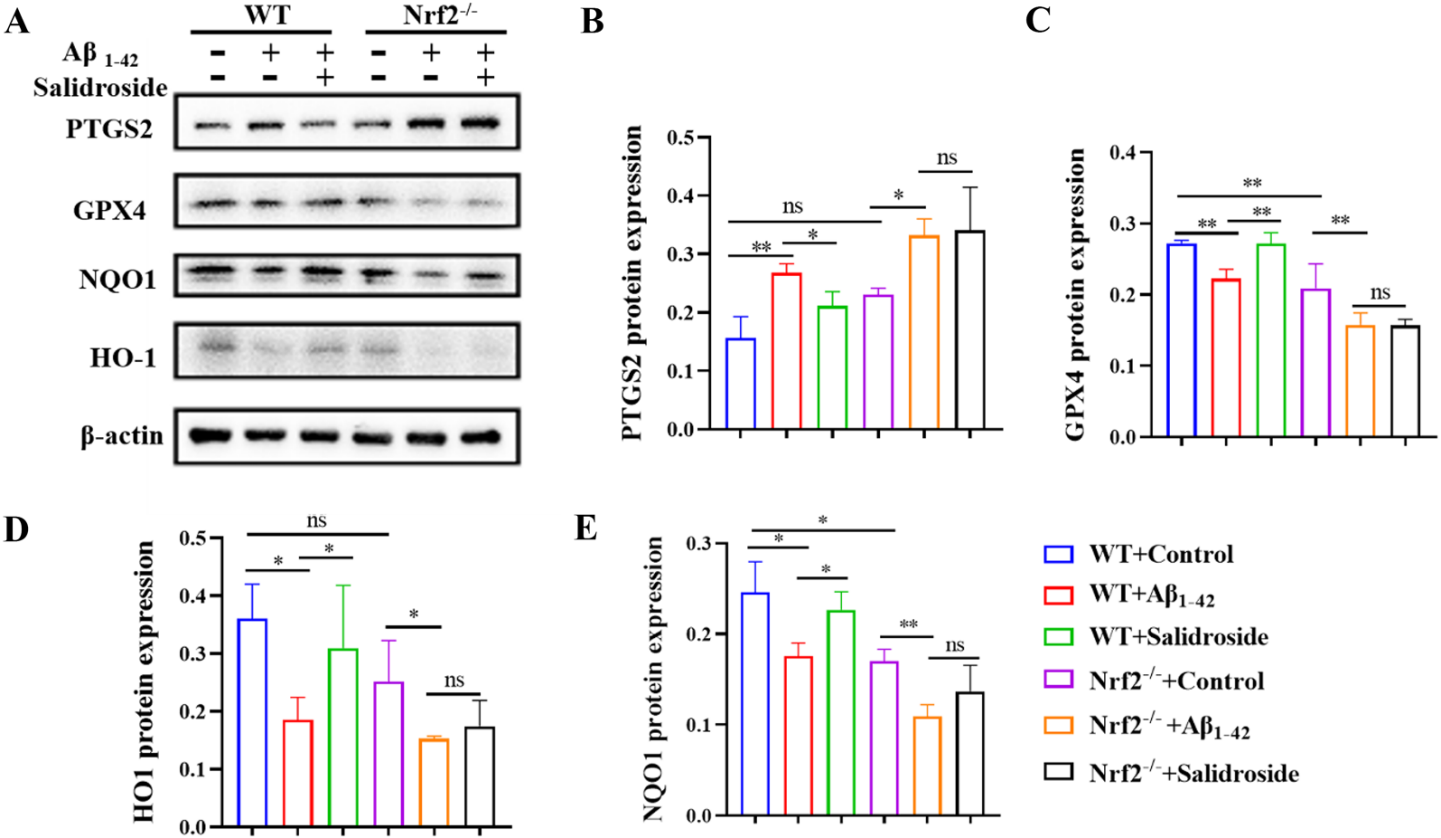
Salidroside regulated the expression of ferroptosis-related proteins in WT mice, but not in Nrf2^−/−^mice. Salidroside regulated the expression of GPX4, PTGS2, Nrf2 downstream antioxidant proteins NQO1, HO1 in hippocampus of WT mice, but it had no significant effects on Nrf2^−/−^ mice. (n = 3 for each group), *P < 0.05, **P < 0.01, ***P < 0.001, “ns” means no significant difference (*P* > 0.05)

GPX4 is the key protein of ferroptosis, and immunohistochemistry was performed to detect its expression in the hippocampus and cortex. To explore whether Nrf2 activation contributes to neuroprotection by treatment with salidroside, we detected and contrasted the expression of Nrf2 target proteins and ferroptosis-related proteins between WT and Nrf2^−/−^ mice by Western blot. Salidroside alleviated the decrease in GPX4 caused by Aβ_1−42_ in the hippocampus and cortex, but this change was not observed in Nrf2^−/−^ mice (Figs. 8B and 9A, C). The results indicated that, compared to the Aβ_1−42_ group, the Nrf2 target antioxidant proteins NQO1 and HO1 were significantly upregulated in the salidroside group of WT mice, but more importantly, not in Nrf2^−/−^ mice (Fig. 9A, D, E). These results supported the vital role of the Nrf2-dependent pathway underlying the neuroprotection of salidroside. Compared with the Aβ_1−42_ group, the ferroptosis-related protein PTGS2 was significantly downregulated in WT mice but not in Nrf2^−/−^ mice treated with salidroside (Fig. 9).

## Discussion

The present study demonstrates that salidroside has neuroprotective effects in Aβ_1−42_-induced WT mice or Glu-injured HT22 cells but not in Nrf2^−/−^mice or siRNA Nrf2 transfected HT22 cells, indicated the activation of the Nrf2/HO1 pathways was involved in salidroside neuroprotection. Salidroside reduced the levels of lipid peroxidation and ROS in Glu-injured HT22 cells but had no significant effects on Nrf2 siRNA transfected HT22 cells. WT mice but not Nrf2^−/−^ mice treated with salidroside exhibited increased expression levels of GPX4. The siRNA Nrf2 transfection experiment also obtained the same results. These results suggested that salidroside alleviated ferroptosis in Aβ_1−42_-induced AD mice and Glu-injured HT22 cells by activating the Nrf2/HO1 signaling pathway.

Recent studies have shown that salidroside inhibited inflammation and apoptosis, promoted dendritic and synaptic plasticity [38], inhibited pyroptosis [39], inhibited oxidative stress, and promoted mitophagy [24]. Accumulated evidence indicated that extracts of *Rhodiola Rosea L.*or salidroside reversed DNA damage and altered the expression of cytokines and antioxidative enzymes induced by ROS [40]. Studies have shown that salidroside can reverse hypoxia injury in PC12 cells by mitochondrial protection via inhibiting oxidative stress events [41], salidroside promoted telomerase reverse transcriptase protein expression via the PI3K/Akt pathway, and maybe a therapeutic strategy for aging-related disease treatment [42]. Our results showed that salidroside increased the viability of Glu-injured HT22 cells and alleviate the cognitive impairment induced by Aβ_1−42_. The results also showed the neuroprotective effects of salidroside.

Aberrant activation of programmed cell death pathways was involved in the processes of neurodegenerative diseases, and the characteristics of ferroptosis, such as iron and lipid peroxide accumulation, are consistent with several hallmarks of AD pathogenesis. Ferroptosis is thought to be a distinct mechanism of cell death in the pathogenesis of AD [43]. Metabolic enzymes implicated in phospholipid peroxidation require iron for catalysis, and iron is also essential for a plethora of metabolic enzymes involved in the generation of cellular ROS.

The nonenzymatic, iron-dependent Fenton chain reaction is likely essential for ferroptosis: when GPX4 is inhibited, phospholipid hydroperoxides (PL-OOH) can persist longer, initiating the Fenton reaction to rapidly amplify PL-OOH, the hallmark of ferroptosis [44]. PL-OOH can react with both ferrous and ferric ions to generate the free radicals PLO• and PLOO•, respectively, and these free radicals react with phospholipids (PUFA-PLs) to further propagate PL-OOH production. Glutamate induces the accumulation of ferrous irons in HT22 cells, which has been confirmed in previous studies [36].

In this study, we investigated whether the neuroprotective effect of salidroside was related to a reduction in neuronal ferroptosis. We found that salidroside restored Glu-injured HT22 cell viability and morphology. Salidroside improved the morphology of mitochondria, increased the mitochondrial membrane potential, decreased the level of ROS and lipid peroxidation, increased the content of SOD and GSH, decreased the content of MDA, reduced the content of ferrous iron, and increased the level of SLC7A11 and GPX4. These findings suggested that salidroside attenuated Glu-induced ferroptosis in HT22 cells. In this study, after treatment with salidroside, cognitive function was improved in Aβ_1−42_-induced AD mice, the ferroptosis-related protein GPX4 was enhanced, and the level of PTGS2 was decreased in WT mice but not in Nrf2^−/−^ mice. These results indicated that salidroside can play a neuroprotective role by reducing ferroptosis in neurons.

Multiple pathogenic processes were mitigated by Nrf2 activation, and the mechanism included improvement of antioxidant defenses and upregulation of mitochondrial function. The elevation of Nrf2 appears to be relatively mild and insufficient to prevent neuronal dysfunction [45]. In AD brains, decreased expression of Nrf2 and its downstream genes have been observed. The key processes by which Nrf2 attenuates AD include interference with the Aβ and p-tau pathways [46]. Our results showed that the expression of NQO1 and GPX4 proteins in Nrf2^−/−^ mice was decreased, which suggested that Nrf2 knockout decreased the expression of downstream antioxidant gene proteins and GPX4 protein. Although there was no significant difference in the expression of HO1 protein and PTGS2 protein between Nrf2^−/−^ mice and WT mice. These results suggested that Nrf2 knockout made mice more susceptible to ferroptosis.

Salidroside is a natural phenolic compound. Phenolic compounds, such as chlorogenic acid [47], honokiol [48], and hydroxytyrosol [49], are well known to have the ability to activate Nrf2 via their redox cycling intermediates. A recent study indicated that salidroside could protect against osteoporosis by inhibiting oxidative stress through the up-regulating of Nrf2 [50]. Salidroside also showed anti-steatotic effects by activating hepatic Nrf2 and increasing the activity of AMPK [51]. We speculated that the neuroprotective effect of salidroside may be related to the activation of the Nrf2 signaling pathways. Based on the important role of Nrf2 in the pathogenesis of AD, this study investigated whether salidroside could alleviate ferroptosis in HT22 cells and AD mice by regulating Nrf2.

Nrf2 is a transcriptional master regulator of neuronal resistance to oxidative stress and Glu-injured excitotoxicity in the nervous system [52]. Nrf2 accumulation to facilitate binding to the ARE requires decomposition of the Keap1/Nrf2 complex. Under conditions of oxidative stress, ROS modify the structure of Keap1, which is a thiol-rich protein with many cysteine residues that is vulnerable to attack by electrophiles, increasing free Nrf2 levels in the cytoplasm. Nrf2 stabilization is involved in the recovery of cellular antioxidant defenses via the upregulation of antioxidant enzymes and proteins, an increase in redox transport, and the induction of stress response proteins, such as HO1 [53].

HO1 is an Nrf2-dependent gene that is highly upregulated following lethal stimuli, such as oxidative stress [15]. A study showed that Glu can increase the levels of Nrf2 and HO1 in a time-dependent manner [37]. In this study, the results suggested that salidroside can inhibit ferroptosis in HT22 cells by increasing Nrf2 expression and nuclear translocation. However, the effects of salidroside on reducing lipid peroxidation and ROS production and upregulating ferroptosis-related proteins were not observed after siRNA Nrf2 transfection in HT22 cells. No improvement in cognitive behavior or regulation of ferroptosis-related proteins was observed in Nrf2^−/−^ AD mice. These results revealed the Nrf2-dependent neuroprotective effects of salidroside. A previous study showed that salidroside can exert an anticonvulsant and neuroprotective effect by activating the Nrf2/ARE signaling pathway [28], the activation effect of salidroside on Nrf2 can also be consistent with our present study.

Our results provided pharmacological basis for further developing salidroside as a therapeutic drug for AD. However, there were some limitations in the present study. Firstly, many upstream signaling pathways of Nrf2, such as MAPK and PI3K/AKT, have been proposed. Further studies are needed to determine whether salidroside activates Nrf2 through these signaling pathways in AD and how salidroside activates Nrf2. Secondly, whether the neuroprotective effect of salidroside in AD contributes to other properties, such as anti-inflammation, should be clarified. We plan to next investigate the upstream signaling pathways and explore whether their effect on reducing ferroptosis in neurons coexists with other neuroprotective effects and what their interaction is.

## Conclusions

In conclusion, salidroside plays a neuroprotective role by alleviating neuronal ferroptosis in Aβ_1−42_-induced AD mice and Glu-injured HT22 cells, and its mechanism is related to activation of the Nrf2/HO1 signaling pathway.

## Data Availability

The data analyzed during this study can be obtained from the corresponding author on reasonable request.

## Abbreviations

AD: Alzheimer’s disease
Aβ: Amyloid β
Nrf2: Nuclear factor E2-related factor 2
Glu: Glutamate.
Fer-1: Ferrostatin-1
HO1: Heme oxygenase1.
SLC7A11: Solute carrier family 7 membranes 11
GPX4: Glutathione peroxidase 4
DCFH-DH: 2′,7′-dichlorofluorescin diacetate
GSH: Glutathione
GSSG: Oxidized glutathione disulfide
SOD: Superoxide dismutase
MDA: Malondialdehyde
JC-1: 5,5′,6,6′-tetrachloro-1,1′,3,3′-tetraethyl-imidacarbocyanine iodide
MMP: Mitochondrial membrane potential
ROS: Reactive oxygen species
